# Impact of acupuncture on otoacoustic emissions in patients with tinnitus

**DOI:** 10.1016/S1808-8694(15)30119-1

**Published:** 2015-10-19

**Authors:** Renata Frasson de Azevedo, Brasilia Maria Chiari, Daniel Mochida Okada, Ektor Tsuneo Onishi

**Affiliations:** a Master’s degree in sciences, UNIFESP - Discipline of Human Communication Disorders, Speech Therapy Department. Speech therapist; b Full professor in the Discipline of Human Communication Disorders, Speech Therapy Department, UNIFESP; c Specialization in Otology, UNIFESP. Otorhinolaryngologist and acupuncturist; d Doctor in medicine, UNIFESP-EPM. Head preceptor in the residency program and coordinator of the Tinnitus Outpatient Unit, Otorhinolaryngology and Head & Neck Surgery Department, UNIFESP-EPM. Sao Paulo Federal University

**Keywords:** acupuncture, otoacoustic emissions, tinnitus

## Abstract

The treatment of tinnitus is still a challenge. Acupuncture is recommended for the relief of tinnitus in traditional Chinese Medicine, although scientific evidence is lacking. **Aim:** The aim of this study was to assess the effect of acupuncture on the cochlear function in patients with tinnitus by analyzing otoacoustic emissions. **Methods:** Thirty eight patients with tinnitus were included in the prospective clinical study. Measures of transitory otoacoustic emissions and suppression of otoacoustic emissions were obtained from all subjects before and after acupuncture. Patients were assigned to one of two groups: intervention group 1 (n=19), in which needle acupuncture was applied at the temporoparietal point corresponding to the vestibulocochlear area, and intervention group 2 (n=19), in which the needle was applied 3cm cranially to this area (which is not a recognized acupuncture point). **Results:** There was a significant difference between the amplitude of otoacoustic emissions assessed before and after acupuncture in intervention group 1. No difference was observed in intervention group 2. **Conclusion:** Acupuncture had a significant effect on otoacoustic emissions in patients with tinnitus.

## INTRODUCTION

Tinnitus is defined as a sensation of sound perceived by a person regardless of an external stimulus. It is generally compared to the sound of hissing, of a whistle or a waterfall.^1^, ^2^

Studies have shown that the prevalence may reach 32% of individuals;^3^ in 0.5% of individuals, symptoms are so severe that normal life is impossible.^4^

The heterogeneity of the population of persons with tinnitus had given rise to many hypotheses to explain its origin.^5^ There are a number of models that have tried to explain the mechanism of tinnitus. Most of the models have suggested some form of cochlear dysfunction.^6^, ^7^, ^8^, ^9^ The hypothesis of discordant damage suggests that tinnitus is generated on the portion of the basilar membrane where there is preservation of inner hair cells (IHC) but damaged or temporarily dysfunctional outer hair cells (OHC). This theory may explain the occurrence of tinnitus in normal hearing individuals, as diffuse damage of up to 30% of OHC can occur without any associated detectable hearing loss.^6^ Another theory is that there would be reduced efferent inhibition in areas where IHC are damaged. Consequently, active basilar membrane areas would increase, causing tinnitus.^7^

A biochemical model for tinnitus has suggested that endogenous dynorphins potentialize excitatory glutamate activity in cochlear NMDA (n-metil-d aspartate) receptors, thereby potentializing elevated auditory system levels. This action would cause a synchronous auditory neuronal discharge in silence, which would be perceived as real sound.^10^

A neurophysiological model described in 1993 states that the main difficulties found in the assessment and treatment of tinnitus are, as follows:

1- the difficulty of objectively examining tinnitus,
2- tinnitus is a symptom that can be associated with a huge number of central and peripheral conditions,
3- lack of proof of the mechanisms for generating tinnitus,
4- tinnitus causes a significant impact on the patient’s central nervous system, and
5- there is a strong connection between the perception of tinnitus and the emotional system.^11^

The appearance of tinnitus is so diverse that the treatment of this symptom is still a significant challenge, although various methods have been successfully used. Acupuncture is one of these methods.^12^, ^13^ In traditional Chinese medicine acupuncture is recommended for the relief of tinnitus, although scientific proof is lacking. Studies have demonstrated that needle stimulus introduces an electrical charge that sets action potentials in motion to rebalance the system.^14^, ^15^

Most of the studies on the efficacy of acupuncture lack objective data to demonstrate the improvement reported by patients. Studies tend to use the patient’s reference of a subjective sensation to analyze the effect of treatment when dealing with tinnitus.^16^, ^17^ A systematic review of the efficacy of acupuncture in the treatment of tinnitus concluded that the belief that acupuncture is effective for treating tinnitus is not based on evidence from controlled, randomized studies.^18^

Auditory assessment has progressed and may provide help in evaluating and monitoring tinnitus patients before and after treatment.

One of the most significant alternatives to study cochlear function in humans is the investigation of otoacoustic emissions (OAE).^19^

OAE are sounds spontaneously generated within the normal cochlea or in response to acoustic stimulation. It is thought that OAE reflect active biological mechanisms within the cochlea and that OHC are responsible for these mechanisms.^20^

The mechanical movement of OHC in the cochlea is probably controlled by efferent auditory pathways. It is thought that the efferent system modulates OHC movement through the medial olivocochlear tract by releasing acetylcholine into synapses.

A simple method to investigate the operation of this system is to compare the amplitude of transient OAE (TOAE) with and with no contralateral auditory stimulation, the measurement of OAE suppression. In normal working efferent auditory pathways, the OAE response amplitude is reduced when a contralateral stimulus is presented.^21^ These effects has been attributed to the action of the medial olivocochlear tract on OHC synapses, attenuating cochlear amplification gain and reducing cochlear membrane movement. The resulting effect is a change in the amplitude of OAE.^22^ The importance of analyzing the effect of TOAE suppression in assessing the influence of cochlear efferent neural activity has been reported in the literature.^23^, ^24^ Efferent auditory pathway dysfunction may be involved in the production and auditory perception of tinnitus. It is thought that dysfunction of the efferent system leads to loss of modulation of OHC, generating abnormal auditory pathway activity that could be erroneously perceived as sound. Investigation of tinnitus patients has shown less effective suppression compared to individuals that do not complain of tinnitus.^23^, ^25^, ^26^

This study, therefore, aimed to analyze the hypothesis that acupuncture causes changes in cochlear function in tinnitus patients.

## OBJECTIVES

The aim of this study was to investigate the effect of acupuncture on the cochlear function of tinnitus patients by using TOAE and measurements of TOAE suppression.

## MATERIAL AND METHODS

### Analysis by the ethics committee

This study was approved by the Research Ethics Committee of the Sao Paulo Hospital (Hospital Sao Paulo number 1079/04).

### Inclusion criteria

Tinnitus patients with auditory thresholds below or equal to 25 dBNA from 250 to 2000Hz and a bilateral type A tympanometric curve.

### Data collection

Subjects were selected among patients routinely attended at the Tinnitus Outpatient Unit of the Otorhinolaryngology and Head & Neck Surgery Department of the Paulista Medical School, Sao Paulo Federal University (UNIFESP-EPM). After explanations about the study, patients chose whether to participate or not. Upon agreement, patients signed a free informed consent form that had previously been approved by the UNIFESP Research Ethics Committee.

### Series

Thirty-eight patients (25 women and 13 men) aged between 36 and 76 years were included in this study.

### Procedures

Preliminary OAE recording

This study was a prospective double-blind trial. Patients initially underwent an auditory evaluation composed of the following tests:

a. TOAE using an ILO 292 device.

The test started with the checkfit to verify the probe in the patient’s external acoustic canal. After probe adjustment the test was started in quickscreen mode.

Recording used non-linear clicks at regular 80 microsecond pulses, rarefaction polarity and repetition frequency of 50 cycles per second. Series of 260 stimuli were computed in 8-click blocks for each test, according to the non-linear technique. The intensity was 80 dB 3 dBpeSPL.

b. TOAE suppression. TOAE amplitudes collected according to the procedure described above, with and without contralateral white noise (50 dBSPL) emitted by an audiometer through a contralateral TDH-39 earphone, were compared to measure the suppression effect contralateral.

### Division of groups and application of acupuncture

After the auditory evaluation, the otorhinolaryngologist-acupuncturist separated the patients into two groups alternatively according to the order of attendance in the clinic.

Patients were accommodated in a silent room and acupuncture needling was done on the side where tinnitus was reported; if tinnitus was bilateral, needling was done in the side where tinnitus was more intense.

**Figure 1:**
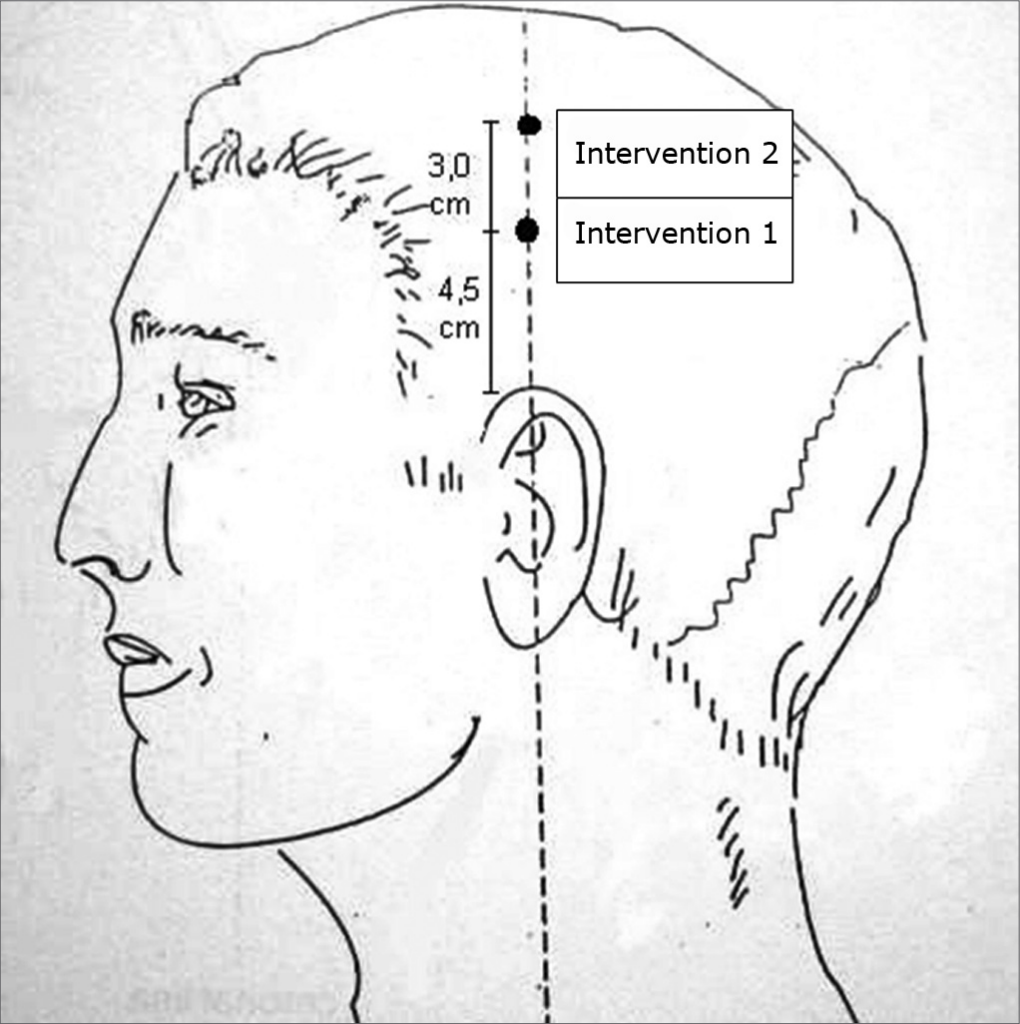
Acupuncture points used in both groups - Intervention 2 / Intervention 1

The acupuncture point used in the intervention 1 group was located 4.5cm above the apex of the ear pinna in the temporoparietal region; this point was found with an acupuncture point locator and relates to the cochleovestibular area in the craniopuncture technique. The acupuncture point used in the intervention 2 group was located 3cm above the previous point along the same vertical line, where the acupuncture point locator emitted no characteristic signal, therefore indicating no acupuncture point at this spot (Figure 1). Disposable stainless steel 0.3x40mm needles were used, which were introduced into the scalp at a 45° angle until reaching the periosteum. Rotating manual stimulation was done at 2 Hz for 15 seconds after which patients remained in silence for one minute.

### OAE measurement after acupuncture

After the procedure described above, patients underwent auditory testing for TOAE and TOAE suppression. The professional that evaluated the patients did not know to which group (1 or 2) they belonged. The second test was done in the same day, 10 minutes after the acupuncture session. Data were fed into a computer spreadsheet for statistical analysis.

### Statistical data analysis

The variables OAE amplitude by a transient stimulus and OAE suppression before and after acupuncture were studied statistically by the analysis of variance (ANOVA) method, a parametric test for comparing the means based on variance. The significance level was 0.05.

## RESULTS

### Characterization of the sample

The sample was composed of 38 subjects with a complaint of tinnitus. The following data is the descriptive analysis of the sample.

**Table 2:** Descriptive statistics of the variable age in each group.

Age	Intervention 1	Intervention 2
Mean	55.05263	57.10526
SD	11.76848	9.194773
median	56	58
maximum	76	68
minimum	38	36
size	19	19

### OAE values

Descriptive statistics of OAE before and after the acupuncture session are shown on Table 3. Groups were divided according to the side acupuncture was applied, side of the ear and moment when emissions were collected (before or after acupuncture) for the statistical analysis.

**Table 3:** Descriptive statistics of amplitudes in decibel sound pressure level (dBSPL) of OAE according to the group, the side acupuncture was applied, the ear and the moment.

Group	Acupuncture side	Ear	Moment	n	Mean	Standard Deviation
Intervention 1	Left	Right	Before	8	6.70	5.79
			After		8.15	6.07
		Left	Before	8	4.18	5.03
			After		5.46	4.85
	Right	Right	Before	11	6.25	5.93
			After		7.20	5.66
		Left	Before	11	6.17	4.80
			After		6.65	4.70
Intervention 2	Left	Right	Before	10	4.96	4.08
			After		4.76	4.12
		Left	Before	10	4.08	5.32
			After		4.11	5.15
	Right	Right	Before	9	3.72	4.23
			After		4.04	3.77
		Left	Before	9	5.08	4.68
			After		5.09	3.90

### Statistical analysis of OAE results collected before and after acupuncture

The ANOVA test was used for the statistical analysis. The mean profile charts indicate mean values for both groups and a standard error (SE) before and after the intervention. The standard error is a measurement of the variability between sample means where a higher standard error indicates a higher variability of the means.

Results of OAE amplitudes collected before and after the intervention in both study groups.

### Right sided acupuncture and analysis of the effect on the right ear

**Chart 1:**
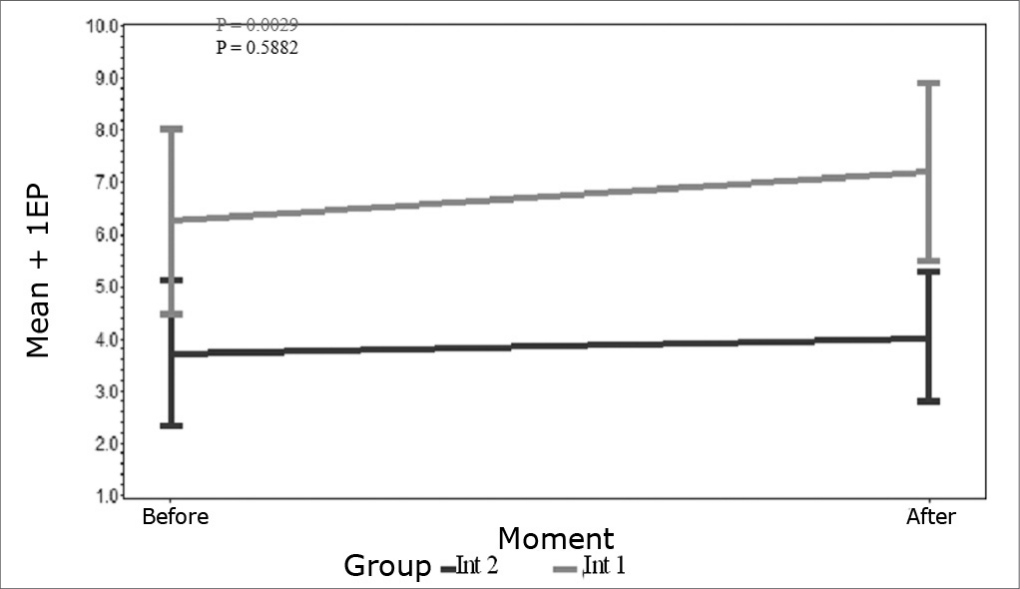
Mean profile of the amplitude of OAE in right ears in right sided acupuncture - key on the chart.

In the intervention 1 group there was a statistically significant difference in OAE collected before and after each acupuncture session. After the acupuncture session OAE were increased (p=0.0029) as shown in Chart 1. In the intervention 2 group there was no statistically significant difference in OAE before and after the acupuncture session (p = 0.5882); in other words, in the intervention 2 group emissions before the acupuncture session did not differ from emissions after the session.

### Left sided acupuncture and analysis of the effect on the left ear

**Chart 2:**
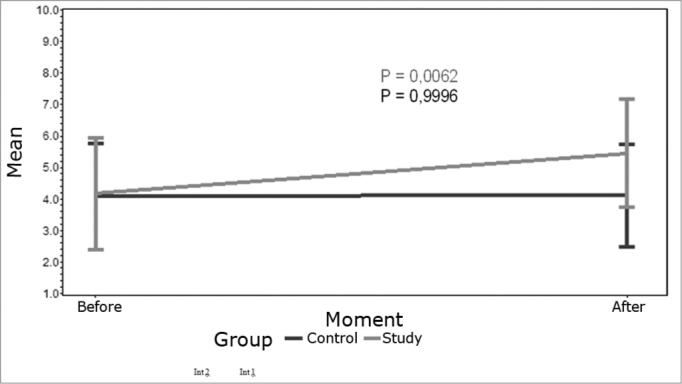
Mean profile of OAE in left ears before and after left sided acupuncture - key on the chart.

In the intervention 1 group there was a statistically significant difference in OAE collected before and after the acupuncture session. After the acupuncture session OAE were increased (p=0.0062) as shown in Chart 2. In the intervention 2 group there was no statistically significant difference in OAE before and after the acupuncture session (p = 0.9996), in other words, in the intervention 2 group emissions before the acupuncture session did not differ from emissions after the session.

### Left sided acupuncture and analysis of the effect on the right ear (contralateral)

In the intervention 1 group there was a statistically significant difference in OAE collected before and after the acupuncture session (p = 0.0327). In the intervention 2 group there was no statistically significant difference in OAE before and after the acupuncture session (p = 0.9644), in other words, in the intervention 2 group emissions before the acupuncture session did not differ from emissions after the session.

### Right sided acupuncture and analysis of the effect on the left ear (contralateral)

In the intervention 1 group there was no statistically significant difference in OAE before and after the acupuncture session (p = 0.4926).

In the intervention 2 group there was also no statistically significant difference in OAE before and after the acupuncture session (p = 1.0000), in other words, in the intervention 2 group emissions before the acupuncture session did not differ from emissions after the session.

### Suppression

Table 4 shows the descriptive statistics of suppression. The intervention 1 group had higher suppression means after the acupuncture session. Mean amplitude suppression values are presented in dBSPL (decibel sound pressure level)

**Table 4:** Descriptive statistics of suppression according to the group, acupuncture side, ear and moment.

Group	Acupuncture side	Ear	Moment	n	Mean	Standard Deviation
Intervention 1	Left	Right	Before	8	0.74	1.25
			After	8	1.11	1.53
		Left	Before	8	0.61	0.84
			After	8	1.34	0.88
	Right	Right	Before	11	0.75	0.67
			After	11	1.16	1.12
		Left	Before	11	1.21	1.09
			After	11	1.35	1.19
Intervention 2	Left	Right	Before	10	1.08	0.95
			After	10	0.85	0.76
		Left	Before	10	0.68	0.86
			After	10	0.60	0.74
	Right	Right	Before	9	1.11	1.13
			After	9	1.37	1.11
		Left	Before	9	1.37	0.71
			After	9	1.29	0.81

The statistical results for the analysis of suppression amplitude are presented below for each group after the intervention.

### Analysis of suppression of the right ear after right sided acupuncture.

In the intervention 1 group there was no statistically significant difference in suppression before and after the acupuncture session (p = 0.3251). The same applies to the intervention 2 group (p = 0.7681)

### Analysis of suppression of the left ear after right sided acupuncture (contralateral)

In the intervention 1 group there was no statistically significant difference in suppression before and after the acupuncture session (p = 0.5582), in other words, right ear acupuncture did not significantly alter contralateral suppression. The same applies to the intervention 2 group (p = 0.7992)

### Analysis of suppression of the right ear after left sided acupuncture (contralateral)

In the intervention 1 group there was no statistically significant difference in suppression before and after the acupuncture session (p = 0.8524), in other words, left sided acupuncture did not significantly alter right sided suppression. The same applies to the intervention 2 group (p = 0.9795).

### Analysis of suppression of the left ear after left sided acupuncture.

**Chart 3:**
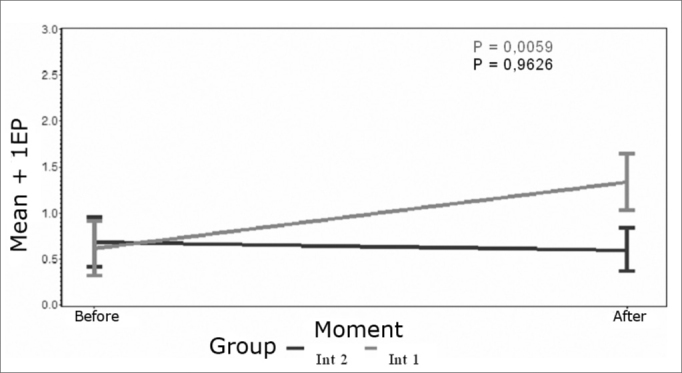
Mean profile of left ear suppression after left sided acupuncture - key on the chart.

In the intervention 1 group there was a statistically significant difference in suppression before and after the left sided acupuncture session (p = 0.0059). In the intervention 2 group acupuncture did not alter suppression (p = 0.9626) as shown on Chart 3.

## DISCUSSION

Tinnitus is undoubtedly one of the main otological findings in the practice of medicine.2 Although there have been many studies on this subject there is still no definitive treatment for this symptom. Tinnitus is frequently considered an idiopathic condition. It causes significant discomfort, at times leading to depression, irritability and even suicide attempts.5,6 This symptom, however, is as subjective as pain; it is still impossible to measure with exams and has not yet been fully understood by researchers. It is a symptom with many probable causes, many mechanisms and much confusion in its treatment. This situation increases stress in tinnitus patients, who end up believing that nothing can be done for them. A common approach is for health professionals to send these patients home after stating that nothing can be done and that they have to learn to live with the symptom.^11^ Many studies are currently being conducted on this subject. A neurophysiological model underlines the importance of the limbic system in emotionally reinforcing tinnitus, which may be responsible for maintaining the perception of tinnitus.^7^ We believe that the persistence and effect of tinnitus on a person’s quality of life is related to the patient’s emotional and physical balance patterns. Health professionals that deal with these patients should provide special care and be patient and persistent. Patients should be reassured that there are various alternatives for symptomatic relief and that their participation is essential for success.

Acupuncture stimuli in specific points seek to evoke a response from the organism to resolve a specific clinical situation, to recover health or to prevent disease. This result is due to an incremental stimulation of regenerative processes, normalization of organic regulatory and control functions, modulation of immunity, promotion of analgesia and harmonization of endocrine, autonomous and mental functions.^12^

Acupuncture in Brazil has been recognized as a medical specialty by the Brazilian Medical Association (AMB) since 1995. It is gaining popularity in academia, and has become part of the curriculum of some medical graduation courses.

This study strengthens the scientific evidence for acupuncture as a therapy for relieving tinnitus. We used OAE testing given its simplicity, speed, objectiveness, noninvasive nature and importance in assessing cochlear and efferent olivocochlear system function,^19^ both of which are relevant systems involved in generating tinnitus, according to most of the current hypotheses.

This study showed a higher prevalence of women in both groups (68% of the intervention 1 group and 63% of the intervention 2) (Chart 1). An epidemiological survey done in the Hospital das Clinicas (Clinical Hospital) found that 60% of patients were women.1 Another study has also reported a higher prevalence of tinnitus in women, in persons exposed to noise and in the lower income bracket population.^4^ There are still other explanations for an increased prevalence in women. On average women live longer than men and therefore are more likely to develop chronic diseases such as diabetes and high blood pressure and their complications, as well as presbyacusis and tinnitus.

The mean age in the intervention 1 group was 55 years, ranging from 38 to 76 years. The mean age in the intervention 2 group was 57 years, ranging from 36 to 68 years (Table 1). These numbers are similar to those found in the literature, which shows that tinnitus is a symptom the prevalence of which increases with age, reaching about one third of the population over 65 years.^11^ Other studies have also reported a significantly increased number of patients as age progresses.^3^, ^4^

**Table 1:** Distribution of the sample into the study groups according to sex.

	Male	Female	Total
Intervention 1	6	13	19
Intervention 2	7	12	19

Total	13	25	38

Many acupuncture studies have used subjective data to assess its effect, such as scores to assess symptom improvement.^16^, ^17^

Axelsson et al. (1994) studied the efficacy of acupuncture in tinnitus patients by using a visual analog scale. This study showed no significant difference.17 Park (2000) noted the lack of evidence-based data from randomized trials in this area, having suggested that further studies should be made using adequate methodologies.^18^

The analysis of OAE amplitude data collected on the right from the right ear before and after acupuncture showed a significantly higher OAE value in the intervention 1 group patients after the acupuncture session, a finding that was not seen in the group of patients subjected to “control” acupuncture (intervention 2 group). Based on this datum we may suggest that acupuncture had an effect on hair cells that resulted in increased amplitude of OAE. Analysis of the effect of acupuncture on the left ear of patients with worse tinnitus in the left ear showed a statistically significant difference in OAE before and after acupuncture (Chart 5). After the acupuncture session, OAE were increased in the intervention 1 group. In this case, we may also consider that acupuncture had an effect on the inner ear.

OAE reveal the integrity of cochlear function; studies have shown that response amplitude is proportional to the number of intact cells and the cochlear function. Studies on the generation of tinnitus associate cochlear damage to the onset of tinnitus.^5^, ^6^, ^7^, ^9^, ^11^ OAE testing analyzes the function of OHC and the active cochlear mechanisms;^20^ a perceived change in the amplitude of emissions after the acupuncture session may indicate that there was an effect on cochlear function. About the generation of tinnitus, we may infer that acupuncture corrected a dysfunction of the peripheral auditory system in some way.

Acupuncture points have a lower electrical resistance. Acupuncture needles accumulate electrical charges on their tips, which generate a difference in potential.^14^ Upon needle stimulation, this difference produces an electrical charge that initiate action potentials, which balance the organism.^15^ The expected action of acupuncture is to balance and harmonize the function of an organism, to reestablish dynamic equilibrium. A published paper has shown that opioid peptides are released in the central nervous system following acupuncture.^15^ There is evidence of opioid peptides in the cochlea.^10^ Opioid peptides lower the presynaptic inflow of calcium, reducing neurotransmitter release and consequently inhibiting the nervous system.

Chami et al. (2001) conducted a study that was similar to ours and also found increased distortion product OAE in 8 patients assessed before and after 15 acupuncture sessions, and concluded that acupuncture acted on the cochlea, specifically on the contractile activity of OHC. A further conclusion was that acupuncture, if applied correctly, could be considered a significant ally in the treatment of tinnitus patients.^13^

We also sought to assess the effect of acupuncture on the contralateral ear (which was not needled). We found an effect after applying acupuncture on the left ear whereby the amplitude of OAE was increased in both ears. This effect may be explained by the fact that the auditory system contains crossed pathways; thus, acupuncture may also affect the contralateral ear through the medial olivocochlear system.

Another effect we analyzed was suppression of OAE. Many studies on the generation of tinnitus underline the importance of the efferent olivocochlear system.^8^, ^25^, ^26^ The efferent olivocochlear bundle contains two main tracts (medial and lateral). The medial tract is composed of fibers that cross over to the opposite cochlea and connect directly to the OHC; the medial efferent system, therefore, acts over a crossed system to control afferent transmission.^22^ A published paper showed that this system could be assessed by measuring the suppression of OAE, which is characterized by emission amplitude analysis following contralateral stimulation.^21^ Since then various studies have analyzed the effect of suppression on OAE in tinnitus patients.^8^, ^23^, ^24^. In a normal system, the amplitude of emission is expected to decrease following contralateral stimulation, due to the action of the medial olivocochlear system over OHC.

When we compared the effect of acupuncture in this study by analyzing suppression amplitude values before and after interventions, we found a statistically significant difference in OAE results for the left ear. After acupuncture we found increased suppression (p=0.0059) in the intervention 1 group, which was not seen in intervention 2 group. A study we found in the literature we reviewed, that was similar to ours, also demonstrated decreased suppression in tinnitus patients with a statistically significant difference only in the left ear, showing a decreased effectiveness of the efferent medial olivocochlear system in tinnitus patients.^23^

This finding suggests that acupuncture also acts on the function of the efferent olivocochlear system. The nervous stimulus that results from acupuncture may be responsible for changes in the superior medial olivary complex from which an efferent nervous bundle directed to the Corti organ originates. The acupuncture stimulus may inhibit OHC, leading to a regression of the tinnitus resulting from changes in this structure.^12^

The general analysis of this study underlines the importance of a complete auditory evaluation in tinnitus patients both before therapy and when monitoring the treatment. Acupuncture and other therapies may benefit from the added information on the improvement of cochlear function collected by OAE. This important evaluation tool will provide further data for focused therapy.

In this study only one acupuncture session on a specific cochlear point was done, which was enough to render significant data. It is likely that prolonged acupuncture treatment would be of greater benefit for the relief of tinnitus.

A person’s health depends not only on healthy cells, tissues or organs, but also on a perfect harmony between every function. It is expected that this harmony may be reestablished by using acupuncture.

We hope that acupuncture may be used more widely as a treatment option for tinnitus, and that its use takes place after a complete evaluation of the auditory system in order to monitor improvements.

## CONCLUSION

1. Acupuncture had an effect on TOAE in tinnitus patients.
2. Acupuncture had an effect on the suppression of TOAE in the left ears of tinnitus patients.
